# Reply: Beyond Rhythm Recognition: A Clinical Pathway for Smartwatch-Detected Atrial Fibrillation

**DOI:** 10.1016/j.jacadv.2026.103305

**Published:** 2026-10-06

**Authors:** Yuval Avidan, Baruch Weizman, Margalit Vainerovsky, Sameha Zahra, Stav Brodsky, Mordechai Alperin, Jorge E. Schliamser, Avinoam Shiran, Michael Hauzer, Asaf Danon

**Affiliations:** a Department of Cardiology, Lady Davis Carmel Medical Center, Haifa, Israel; b Ruth and Bruce Rappaport Faculty of Medicine, Technion-Israel Institute of Technology, Haifa, Israel; c Department of Internal Medicine, Lady Davis Carmel Medical Center, Haifa, Israel; d Department of Family Medicine, Meuhedet Health Services, Pardes Hana-Karkur, Israel; e Department of Family Medicine, Clalit Health Services Community Division, Haifa, Israel; f Department of Family Medicine, Faculty of Medicine, Technion-Israel Institute of Technology, Haifa, Israel

We thank the authors for their constructive letter. The central message of our study was straightforward: primary care physicians should be better equipped to interpret patient-generated smartwatch electrocardiograms (ECGs), particularly to distinguish atrial fibrillation (AF) from sinus rhythm.^1^

The authors cite NOAH-AFNET 6 and ARTESiA, which addressed subclinical AF detected by implanted devices, a fundamentally different clinical scenario from a symptomatic patient presenting with a patient-initiated, single-lead smartwatch ECG. In our study, physician interpretation had 74.2% sensitivity and 85.0% specificity for AF, underscoring the potential for both missed and falsely identified AF in clinical practice. Erroneous automated smartwatch classifications were also strongly associated with misinterpretation, raising concern that automation bias may further compound these diagnostic errors. Contemporary guidelines emphasize confirmation of AF and individualized assessment of thromboembolic risk, bleeding risk, and the broader clinical context.^2^ However, this distinction does not diminish the importance of accurate rhythm interpretation. Without an accurate diagnosis, the clinical decision-making process cannot properly begin.

Obviously, a 30-second smartwatch ECG cannot exclude paroxysmal AF, and additional work-up is warranted when clinical suspicion persists, particularly in symptomatic or high-risk patients,^3^ and, in our view, when the tracing is uninterpretable. In such cases, a structured escalation pathway incorporating various forms of longer-term ambulatory ECG monitoring may provide a pragmatic approach to resolving diagnostic uncertainty.

As smartwatch use continues to expand, traditional medical curricula must be modernized to include training on single-lead ECGs. Clinical discussion must move beyond the binary question of whether “AF was detected” toward a more nuanced pathway encompassing confirmation, AF burden, overdiagnosis, and the clinical significance of device-detected episodes.^4^ Ultimately, the goal is not to treat a smartwatch label, but to accurately interpret the recording, recognize uncertainty, and integrate the finding into individualized clinical care. As the old adage reminds us, “treat the patient, not the ECG.”
